# Unsupervised and Supervised Learning over the Energy Landscape for Protein Decoy Selection

**DOI:** 10.3390/biom9100607

**Published:** 2019-10-14

**Authors:** Nasrin Akhter, Gopinath Chennupati, Kazi Lutful Kabir, Hristo Djidjev, Amarda Shehu

**Affiliations:** 1 Department of Computer Science, George Mason University, Fairfax, VA 22030, USA; nakhter3@gmu.edu (N.A.); kkabir@gmu.edu (K.L.K.); 2 Information Sciences (CCS-3) Group, Los Alamos National Laboratory, Los Alamos, NM 87545, USA; djidjev@lanl.gov; 3 Center for Adaptive Human-Machine Partnership, George Mason University, Fairfax, VA 22030, USA; 4 Department of Bioengineering, George Mason University, Fairfax, VA 22030, USA; 5 School of Systems Biology, George Mason University, Fairfax, VA 22030, USA

**Keywords:** energy landscape, basin, decoy selection, machine learning, purity, model quality assessment

## Abstract

The energy landscape that organizes microstates of a molecular system and governs the underlying molecular dynamics exposes the relationship between molecular form/structure, changes to form, and biological activity or function in the cell. However, several challenges stand in the way of leveraging energy landscapes for relating structure and structural dynamics to function. Energy landscapes are high-dimensional, multi-modal, and often overly-rugged. Deep wells or basins in them do not always correspond to stable structural states but are instead the result of inherent inaccuracies in semi-empirical molecular energy functions. Due to these challenges, energetics is typically ignored in computational approaches addressing long-standing central questions in computational biology, such as protein decoy selection. In the latter, the goal is to determine over a possibly large number of computationally-generated three-dimensional structures of a protein those structures that are biologically-active/native. In recent work, we have recast our attention on the protein energy landscape and its role in helping us to advance decoy selection. Here, we summarize some of our successes so far in this direction via unsupervised learning. More importantly, we further advance the argument that the energy landscape holds valuable information to aid and advance the state of protein decoy selection via novel machine learning methodologies that leverage supervised learning. Our focus in this article is on decoy selection for the purpose of a rigorous, quantitative evaluation of how leveraging protein energy landscapes advances an important problem in protein modeling. However, the ideas and concepts presented here are generally useful to make discoveries in studies aiming to relate molecular structure and structural dynamics to function.

## 1. Introduction

Decades of scientific inquiry have demonstrated how fundamental molecular form is to function [1,2]. In particular, protein molecules, which control virtually all processes that maintain and replicate a living cell, harness their three-dimensional (3D) form/structure and changes to structure to regulate interactions with molecular partners [3]. Proteins are intrinsically dynamic and access a vast, high-dimensional structure space. The navigation of a protein’s structure space is regulated by an underlying energy landscape, which organizes structures by their internal (potential) energies [4].

The energy landscape exposes the relationship between molecular structure, changes to structure, and biological activity or function [5]. Characteristics of the landscape are interpretable. Its wells/basins correspond to thermodynamically-stable or semi-stable states (composed of similar structures) that are lived long enough for a molecule to stick to another molecule. Changes between states are regulated by energetic barriers that can be visualized as hills or mountains; these are composed of short-lived, high-energy structures. Having access to the energy landscape allows exposing, in principle, functionally-relevant states as the deep and broad basins. The qualifier “in principle” acknowledges several challenges. Like the structural spaces that they lift by one more dimension (the internal energy of a structure), protein energy landscapes are vast and high-dimensional. They are also multi-modal, containing many local minima. In fact, energy landscapes constructed in silico are overly rugged and contain a multitude of false local minima due to inherent inaccuracies in the semi-empirical energy functions devised to measure the internal energy of a 3D structure [6].

Considering the above challenges, energetics is ignored in many computational methodologies that aim to address long-standing questions in computational biology. One of these is a recognition problem, known as decoy selection [7]. The goal in decoy selection is to determine over a possibly large number of computationally-generated 3D structures of a protein. These structures are biologically-active/near-native. This needle(s)-in-the-haystack problem is exceptionally challenging, but it is a hallmark problem in molecular biology [8].

The motivation for decoy selection comes from the recognition that having access to structure(s) that a protein uses to interact with molecular partners is the first key step towards understanding the array of activities of a protein in the cell. Typically, one would prefer determining these structures in the wet laboratory. However, the great advances in experimental protein structure determination (PSP) techniques have not been able to keep pace with advances in sequencing technologies. These technologies have yielded millions of protein-encoding gene sequences [9]. On the other hand, the number of determined native protein structures is an order of magnitude less. As of July 2019, the number of native structures deposited in the Protein Data Bank (PDB) [10] is 154,015. This discrepancy is at the heart of growing computational efforts. To spur progress in silico, the community leverages a biennial competitive-styled event, the critical assessment of protein structure prediction (CASP) [11].

The most challenging setting in PSP is the template-free one, where target protein sequences with no known structures do not have sufficiently-similar protein sequences with known structures that could otherwise serve as templates [12]. Template-free PSP is carried out in two stages. The first stage generates many structures (decoys) given an amino-acid sequence. The details of decoy generation algorithms are beyond the focus of this paper, but the interested reader is referred to recent reviews in [12,13]. The second stage is what we introduced above as decoy selection, where the goal is to tease out the decoys that are near-native among the thousands or more generated in the first stage.

In Section 1.1, we describe representative work in decoy selection, relating the many advances and remaining challenges. Predominantly, what structure predictors employ relies on unsupervised or supervised learning. In the first category, one finds applications of clustering algorithms that group together geometrically-similar decoys, ignoring their energies. In the second category, one finds applications of supervised learning methodologies, such as support vector machines (SVMs) or neural networks (NNs) that build over many low-level, carefully-curated features.

Despite many challenges, in recent work, we have recast our attention on the role of the protein energy landscape in helping one to advance the state of decoy selection. In this article, we first summarize some of our recent successes in this direction via unsupervised learning. We relate how by leveraging the concept of basins in a methodology that identifies and ranks basins in the energy landscape comprised of thousands of decoys exposes basins rich in near-native decoys [14,15]. We show that utilizing energies yields a distinct, quantifiable improvement over a complementary method that builds over clustering of decoys while ignoring energies [16]. More importantly, we further advance the argument that the energy landscape holds valuable information to advance the state of protein decoy selection via supervised learning. We show how this machine learning-based (ML-based) method improves robustness, yielding good-quality basins even on exceptionally-challenging decoy datasets where clustering-based approaches fail. Finally, we propose a novel method that selects from a given subset of decoys a single, good-quality decoy for prediction. Our methodological contribution is a decoy selection pipeline that first obtains a few basins of high quality and then selects from these basins (and offers as prediction) a decoy of high quality.

It is worth noting that our focus in this article is on decoy selection for the purpose of a rigorous, quantitative evaluation of how leveraging protein energy landscapes advances an important problem in protein modeling. However, the ideas and concepts presented here, particularly those on basin identification and purification, are generally useful to make discoveries in studies aiming to relate molecular structure and structural dynamics to function, such as studies where structures obtained from molecular dynamics simulations need to be organized to summarize the simulated dynamics [17,18]. We believe that, despite its high ruggedness and dimensionality, the energy landscape holds significant information on the inner workings of a molecule and will prove increasingly useful with growing sophistication in algorithmics and hardware.

The rest of the paper is organized as follows. A summary of related work is presented in Section 1.1. The evaluation of the various methodologies that leverage the energy landscape to address decoy selection is related in detail in Section 3. A discussion follows in Section 4. The proposed methodologies are described in Section 2.

### 1.1. Related Work

In the early days, when decoy selection was starting to be recognized as a practical necessity in molecular structural biology, proposed methods aggressively used energies of decoys to determine their “nativeness”. This early enthusiasm, however, soon diminished upon the realization that energy was a poor indicator of nativeness [19]. Many studies reported that lower energy did not relate to closer proximity of a decoy to the native structure [20,21,22]. Consequently, other methodologies became more prominent. Clustering-/consensus-based methods, also known as multi-model methods, dominated the decoy selection category (also known as model accuracy/quality assessment) in CASP [23,24], until recently, when methods based on supervised learning made their debut. Currently, there is great diversity among decoy selection methods. Based on the approach they follow, these methods can be roughly grouped into single-model, multi-model, and quasi-single model methods.

Single-model methods work on a per decoy basis [25] and employ energy functions designed specifically to aid decoy selection. Some of these methods use physics-based functions based on physical properties of atomic interactions [26,27,28]. Others use knowledge-based/statistical scoring functions that rely on statistical analysis of known native structures [29,30,31]. The latter methods have been more successful [32,33]. Clustering-based methods, on the other hand, do not rely on energy or scoring functions. They group together similar decoys and offer the largest *k* clusters as prediction. Some recent work has leveraged concepts, such as communities, from network science to cluster decoys [16]. These methods construct clusters as communities as in social networks.

Until very recently, clustering-based methods decidedly outperformed single-model methods [7]. However, single-model methods have progressed considerably, to the point that they can now compete with clustering-based methods [8]. Since the most successful single-model methods rely on specially-designed scoring functions that users often have to re-implement, clustering-based methods remain more popular. Clustering-based methods pose their own concerns, some of which are addressed in [34,35,36]. Most notably, they suffer from the curse of dimensionality [37] and carry significant computational costs with decoy data of increasing size. Since they are based on consensus, they have a very hard time identifying good decoys in sparse, low-quality decoy datasets, where near-native decoys are significantly under-sampled by decoy generation algorithms.

In the last five years, quasi-single model methods and supervised learning methods have taken hold in the community. These methods currently outperform clustering-based methods. Quasi-single model methods combine concepts of single- and multi-model methods [38,39]. They work by comparing decoys to some selected, high-quality reference structures [40]. Methods based on supervised learning are currently quite diverse, leveraging SVMs [41,42], Random Forest [43], NNs [44,45], and ensemble learning [46]. Feature sets are also diverse, derived from terms of statistical scoring functions [47,48] and/or expert-constructed structural features [49,50]. These methods show great promise.

Inspired by outstanding performance in image recognition, decoy selection research has adopted deep learning strategies. For instance, Cao et al. [45] proposes deepQA, a single-model decoy selection method that utilizes energy, structural, and physio-chemical characteristics of a decoy for quality prediction. Improved decoy selection has also been observed with models based on convolutional neural networks (CNNs). For instance, Hou et al. [51] uses a deep one-dimensional CNN (1DCNN) to build a single-model decoy selection method. The authors make use of two 1DCNNs to predict the local and global quality of a decoy. In [52], the authors propose Ornate, a single-model method that applies a deep three-dimensional CNN (3DCNN) for model quality estimation. 3DCNN has also been used successfully in [53]. Hou et al. observe substantial improvement in protein model selection by using contact distance predicted via a deep CNN [54]. These methods are very promising, but they are still challenged by the scarcity of labeled data, imbalanced data distribution, and more.

## 2. Materials and Methods

This section is organized as follows. We describe in Section 2.1 how one can rigorously adapt and optimize parameter selection for application of the k-means algorithms for decoy selection. We recall that our comparative evaluation of landscape-based methods uses k-means as a baseline; in Section 3, we refer to it as Kmeans-Select. Section 2.2 then summarizes a decoy clustering algorithm based on community detection originally proposed in [16]; in Section 3, we refer to it as Community-Select. The landscape-based method Basins-Select that identifies and offers selected basins for prediction, recently proposed in [14] is summarized in Section 2.3. Section 2.4 then describes ML-Select, which leverages supervised learning to select basins of better quality. Section 2.5 then describes a novel method, Weighted-Decoy-Select, which selects and offers individual decoys for prediction from a given set *S*. We note that the methodological contribution of this article is a pipeline for automatic decoy selection, which first obtains a few basins of high quality via ML-Select and then selects from these basins (and offers as prediction) a decoy of high quality via Weighted-Decoy-Select.

### 2.1. K-Means-Select

The k-means clustering algorithm we employ is implemented in Python’s sklearn library. We optimize two hyper-parameters, the decoys that serve as cluster centroids and the number of clusters *k*. For a given value *k*, the algorithm is initialized with *k* decoys selected uniformly at random over the decoy dataset to serve as the centroids. For a particular grouping *C* of the dataset into *k* clusters, the loss function is measured via the within-cluster scatter: $L\left(C\right)=\frac{1}{2}\sum_{l=1}^{k}\sum_{i\in C_{l}}\sum_{j\in C_{l},j\neq i}D\left(x_{i},x_{j}\right)$, where $D(x_{i},x_{j})$ measures the Euclidean distance between two decoys $x_{i}\neq x_{j}$ in the same cluster $C_{l}$, where l∈{1,…,k}. The algorithm seeks to minimize this loss function via iterative refinement, varying the decoys selected to serve as cluster centroids for a maximum number of iterations, and choosing among the different options the ones yielding the smallest loss. We set the maximum number of iterations to 10 and retaining the cluster centroid assignment resulting in the smallest loss over the iterations.

We determine the optimal value for *k* via the popular knee-finding approach. For a given *k*, after the centroids are determined as above, the squared distance of each decoy in a cluster from that cluster’s centroid is recorded, and the sum of these squared distances/errors (SSE) is then obtained over the clusters *k*. The SSE is plotted for different values of *k*. The knee/elbow in the SSE curve indicates the optimal number of clusters. The SSE approaches zero as one increases *k*; it is exactly 0 when *k* is the size of the decoy dataset. The goal is to choose a small value of *k* that results in a low SSE. The knee in the SSE curve corresponds to the region where increasing *k* yields diminishing returns.

As described in Section 3, the obtained clusters can be ranked based on several characteristics. Size is certainly an intuitive one, with larger size relating to stronger consensus. Thus, clusters can be ordered from largest to smallest size, and the top *x* clusters can be selected and merged into a decoy subset offered as prediction. As related in Section 3, the comparative evaluation of the methods in this paper is limited to x=1 (the largest cluster in this case).

In [14], we propose three other strategies that leverage the size and energy of a group of decoys (with the energy of a group defined as the average or minimum over the energies of the decoys), the Pareto Rank, and the Pareto Rank and Count; the latter two are based on the concept of dominance when comparing groups across several characteristics. The reader is referred to work in [14] for more details. For the purpose of keeping the comparative evaluation focused on the methods that produce the grouping of decoys, we only consider ranking by size.

### 2.2. Community-Select

Recent work in [16] utilizes an on-graph clustering algorithm for the purpose of organizing decoys in clusters or groups. The method relies on two main ideas, embedding decoys in a nearest-neighbor graph (nn-graph) and applying algorithms originally devised to detect communities of users in social networks to group the decoys.

The decoys in a given dataset can be embedded in an nn-graph as follows: The vertex set of the graph is populated with the decoys. The edges are obtained by inferring a local neighborhood structure over each decoy. Using RMSD to compute the distance between two decoys, each vertex *u* is connected to other vertices *v* if d(u,v)≤ϵ, with ϵ being a user-defined parameter. A small ϵ may result in a disconnected graph. This can be remedied by gradually increasing the value of ϵ over a maximum number of iterations, while controlling the density of the resulting nn-graph via a maximum number of nearest neighbors per vertex.

In [16], several community detection algorithms, such as Girvan–Newman, which is based on hierarchical clustering, Leading Eigenvector, which maximizes modularity over communities/clusters, Walktrap, which implements an agglomerative approach, Label Propagation, which seeks a consensus on a unique label for densely-connected vertices, Louvain, which is a heuristic-based method focusing on modularity optimization, InfoMap, which is based on information flow analysis, and Greedy Modularity Maximization, which implements hierarchical agglomeration-based clustering, are compared on several metrics of the clusters/communities they yield, as well as on the purity of top, selected communities. The analysis in [16] concludes that Louvain outperforms the other algorithms. For this reason, the comparative evaluation in Section 3 focuses on evaluating clusters of decoys selected among those identified with the Louvain algorithm over an nn-graph embedding decoys in a given dataset. For further details, as well as analysis on parameter values, the reader is referred to work in [16]. We recall that the evaluation in Section 3 focuses on the largest cluster obtained.

### 2.3. Basins-Select

Basins-Select, recently proposed in [14], additionally takes into consideration decoy energies and identifies groups of decoys that are basins in the underlying landscape from which a decoy generation algorithm has sampled decoys. The method first constructs the nn-graph embedding given decoys, as above, but then identifies basins in the graph by first locating the point of attraction or focal minimum of a basin by identifying vertices that are local (energy) minima in the graph. A vertex is considered a local minimum if its energy is no higher than the energies of its 1-neighbors (other vertices connected to it via an edge). Each local minimum vertex represents a basin. Once local minima vertices are identified, remaining vertices are assigned to basins as follows. Each vertex *u* is associated a negative gradient estimated by selecting the edge (u,v) that maximizes the ratio [e(u)−e(v)]/d(u,v), where e(u) is the energy of the decoy in vertex *u*. From each vertex *u* that is not a local minimum, the negative gradient is followed (via the edge that maximizes the above ratio) until a local minimum is reached. Vertices that reach the same local minimum are then assigned to the basin associated with that minimum. Work in [14] utilizes the Structural Bioinformatics Library (SBL) [55] to decompose a decoy nn-graph into basins. Note that Basins-Select is an unsupervised learning method, like k-means and Community-Select, as the basins it identifies are in essence clusters or groups of decoys. These groups can be ranked via several characteristics for selection. We recall that the evaluation in Section 3 focuses on the largest basin (with most decoys) obtained.

### 2.4. ML-Select

**Figure 1:**
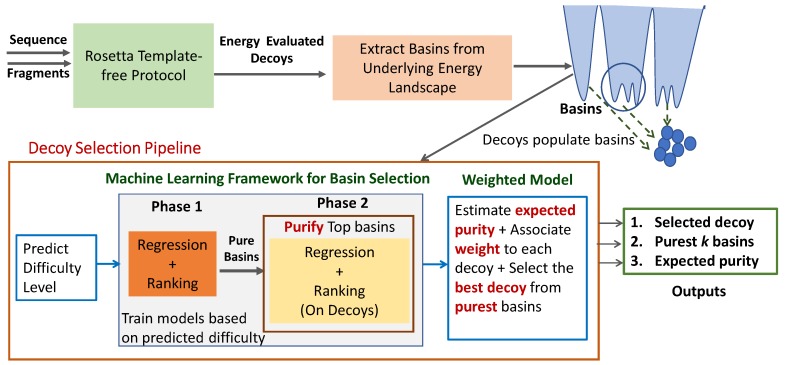
Illustration: Pipeline of ML-Select followed by a weighted model to first select pure basins and then select an individual decoy for prediction.

ML-Select, illustrated in Figure 1, leverages both unsupervised and supervised learning. The unsupervised learning component is the Basins-Select method described above, which groups decoys into basins. Rather than relying on ranking based on simple basin characteristics, ML-Select predicts “best” basins and the “best” decoy from the identified basins via supervised learning in two phases. Figure 1 additionally shows that the pipeline can be extended to permit selection of individual decoys by a weighted model, which we describe in detail in Section 2.5.

**Summary:** As related in Section 2.6, a given decoy dataset can be categorized as *easy, medium, or hard* based on the quality of the best decoy and the percentage of near-native decoys in it. This is possible when the native structure is known, as both metrics (quality of best decoy and percentage of near-native decoys) utilize RMSD from the known native structure. In the absence of a native structure, one can still predict the difficulty of a given decoy dataset via a supervised learning technique that we design and describe below. The reason for such a technique is that optimal prediction of the best basin(s) and best decoy can be achieved on a given decoy dataset if the predictive model is trained on a dataset of similar difficulty level. For this reason, ML-Select first predicts the difficulty level of a given decoy dataset. The resulting information is then passed to Phase 1, which builds a predictive model that selects/predicts the purest *l* basins. These are then passed on to Phase 2. The weighted selection model assigns a weight, also considered as a confidence level, to each decoy in the selected basins. Based on these weights, this module selects the best decoy from the top basin. We now describe each module in greater detail.

#### 2.4.1. Predicting the Difficulty Level of a Given Decoy Dataset

We pose this as a classification task. We have considered two different settings, binary and multi-class classification. In the binary setting, targets are classified as either easy or hard. In multi-class classification setting, targets are either easy, medium, or hard. We hypothesize that the characteristics and peculiarities of the underlying energy landscape probed by a template-free decoy generation algorithm indirectly informs about the difficulty level of a target protein.

The features for the classification models are collected from various measurements associated with basins. We employ four categories of features. The first three are minimum and maximum size of identified basins, minimum and maximum energy over focal minima of basins, and minimum and maximum persistence of basins (persistence relates to the shallowness of a basin [55]). The fourth category is calculated over graph representations of basins. Similar to how decoys of a given dataset are embedded in an nn-graph, the decoys in an identified basin can also be embedded in an nn-graph encoding only that basin. The ϵ parameter is set here to pdist+1Å, where pdist is the average pairwise RMSD between the decoys in a basins. The resulting graph may contain one or more connected components. The frequency of the varying degrees of connected components resulting from the nn-graphs encoding basins is the final feature considered.

The difficulty level of a given decoy dataset is determined according to min_dist and the abundance or scarcity of near-natives available in the decoy ensemble, as related in Section 2.6. A boosting-based ensemble learning approach, XGBoost [56], is trained over thus-labeled datasets and tested on unlabeled decoy datasets to predict the difficulty level of a new decoy dataset. The training dataset comprises two decoy datasets per category (easy, medium, hard). Hence, the size of the training set is approximately 6× 50,000, as the size of each decoy set is approximately 50,000. The trained model is tested over the twelve remaining targets. Decoy sets are selected in turn for training the model to ensure that every target is tested for difficulty prediction.

We note that, although we do not report these experiments in Section 3, we experimented with both binary and multi-class classification problem. The binary classification model correctly classifies all the easy datasets and all but one hard dataset. The multi-class model struggled to classify the medium-difficulty datasets and misclassified 4/5 of the medium-difficulty datasets. However, it correctly classifies all the easy and hard datasets. Overall, our model shows moderate success in classifying the difficulty levels of decoy datasets.

#### 2.4.2. Predicting Basin Purity

Regression is used to predict the purity of a basin in Phase 1 of ML-Select. Specifically, the regression model (XGBoost, see Section 2.6) is trained over identified basins with associated purity values. Two decoy datsets per difficulty level (easy, medium, hard) are selected randomly to train the regression model. The trained model predicts the purity of test decoy dataset. Seven different models, trained over seven different training datasets, are built to ensure that each target is employed in turn as part of a test dataset for predicting its purity. We recall that the purity of a basin is related to precision and is measured as the number of near-native decoys divided by the size of the basin (total number of decoys in it). Two categories of features are employed: Pareto- and graph-based features. The Pareto-based features associated with a basin are its Pareto Rank and Pareto Count calculated using the concept of dominance, comparing basins based on their size and energy. Work in [14] describes the concept of dominance and the calculation of Pareto Rank and Count in greater detail. The graph-based feature associated with each basin is the number of connected components in the nn-graph that encodes the decoys of a basin (computed as described above in Section 2.4.1).

The regression model predicts purity. Basins are then ranked based on the predicted purity (from high to low). The top *n* basins are then passed to Phase 2 of ML-Select for further “purification”.

#### 2.4.3. Purifying Basins

Phase 2 purifies the input *n* basins and offers the top *l* pure basins as outputs. The *n* and *l* parameters are user-defined. Basins are purified based on the predicted RMSD of the decoys populating them. The prediction is performed by a regression model that uses 20 features that are knowledge-based potentials of decoys: RW, RWplus [57,58], dDFIRE [59], and the energy terms in the Rosetta *REF2015* scoring function [60]. A regression model is built for each of the *n* basins provided as input. Model training was performed using the same decoy datasets employed to train the regression model in Phase 1. In a given basin, if the predicted RMSD of a decoy falls short of a pre-defined threshold (dist_thresh), the decoy is removed from the basin. After these eliminations, basins are ranked based on the resulting purity, and the top *l* basins are the output of the second phase. Since the purification process may eliminate a near-native decoy, we mitigate this with a shift in the pre-defined distance threshold, dist_thresh ± τ, where τ ∈ {10%, 20%, 25%} of dist_thresh.

### 2.5. Weighted-Decoy-Select

We note that the result of ML-Select, if terminated after Phase 2, is a subset of decoys consisting of the top, purified, *l* basins. In this respect, ML-Select can be evaluated according to the purity of these basins. The inspiration for offering purity as a metric via which to evaluate the quality of a basin in our earlier work in [14] comes from the recognition that a naive strategy proposed to select an individual decoy for prediction can do so via random uniform sampling over decoys in basin; the higher the purity of a basin, the higher the probability that a decoy selected in this manner is near-native.

The evaluation in Section 3 shows that Basins-Select outperforms clustering-based methods that do not utilize the energy landscape, and that ML-Select further outperforms Basins-Select. However, the following question still stands: provided one narrows the focus to one or a top few basins, how does one select a decoy and offer it for prediction in an intelligent manner? We propose here a weighted selection method, which we refer to as Weighted-Decoy-Select, which utilizes more information than random uniform sampling. Specifically, Weighted-Decoy-Select associates a weight $w_{i}$ with each decoy *i* in a basin *B*, ranks decoys from larger to lower weight, offering for prediction the top decoy in the ranking.

**Weighting Decoys Based on Predicted RMSD:** The fundamental question now relates to how one can define a weight with a decoy. Ideally, higher weight should be given to near-native decoys, and lower weight to non-native ones. As related, the energy of a decoy is not a good indicator of its quality and so cannot be reliably used to define its weight. Instead, we propose that the weight be a function of the predicted RMSD of a decoy. Recall that above, in Section 2.4.3, we introduce a regression model that is trained to predict the RMSD of a decoy. Generally, we then define $w_{i}=f\left(RMSD_{\mathrm{predicted}}\right)$, where *f* is non-increasing, non-negative function. While others can be used, in our evaluation in Section 3, we utilize f(x)=1/(x+ζ), where ζ is some small value protecting against division by 0.

**Weighting Decoys Based on Density:** Alternatively, one can define the weight of a decoy not based on its predicted RMSD, but based on its density. We are inspired here by the density score originally introduced in [61] to evaluate the discriminatory power of a decoy-dependent knowledge-based energy function. The density score $S_{i}$ of a decoy *i* in a set of *m* decoys is defined as $S_{i}=\frac{\sum_{j\neq i}r_{ij}}{m}$, where $r_{ij}$ denotes the pairwise $C_{\alpha }$ RMSD between decoys i,j≠i. As in [61], the density scores are normalized between −1 and 1, and the normalized density score $S_{i}^{{}^{\prime }}$ is computed as:

$$S_{i}^{{}^{\prime }}=\left\{\begin{array}{cc} \frac{\left(S_{i}-S_{\mathrm{median}}\right)}{S_{\mathrm{median}}-S_{\min}}, & \;\mathrm{if}\;S_{i}<S_{\mathrm{median}},\\ 0, & \;\mathrm{if}\;S_{i}=S_{\mathrm{median}},\\ \frac{\left(S_{i}-S_{\mathrm{median}}\right)}{S_{\max}-S_{\mathrm{median}}}, & \;\mathrm{if}\;S_{i}>S_{\mathrm{median}}. \end{array}\right.$$

In the above, the minimum, maximum, and median density scores are denoted by $S_{\min}$, $S_{\max}$, $S_{\mathrm{median}}$, respectively. Finally, based on this normalized density score, we associate with each decoy the following weight $w_{i}=e^{-kS_{i}^{{}^{\prime }}}$, where *k* is a constant that amplifies the impact of the density score on the selection. As in [61], we set k=5 in the evaluation in Section 3. Note that this density-based approach associates lower weight with “outlier” decoys whose distances from other decoys in a basin are large.

**Weighted Purity:** It is important to note that the ability to weight individual decoys in a basin allows for associating a *weighted purity* with a basin as follows: $wp\left(B\right)=\frac{\sum_{i\in B}w_{i}g_{i}}{\sum_{i\in B}w_{i}}$, where the *goodness* $g_{i}$ of a decoy *i* is 1 if *i* is near-native and 0 otherwise. Let *G* be the set of all decoys with $g_{i}=1$. Then, the probability of selecting a near-native decoy is $\sum_{i\in B}p_{i}g_{i}$.

### 2.6. Implementation Details

We collected the amino acid sequences in FASTA format from the PDB [62] and fragment files using the ROBETTA server [63]. The sequence and fragment files were fed to Rosetta template-free ab initio protocol to generate decoys. We used 1Å for the ϵ parameter to build the nn-graph embedding a decoy dataset. The min_dist parameter refers to the minimum distance from a decoy in an ensemble to the native structure deposited to the PDB. We set a distance threshold dist_thresh to determine the near-natives in a decoy dataset. All decoys under the threshold dist_thresh are deemed as near-natives.

The categorization of the easy, medium, and hard cases is governed by the dist_thresh parameter and is done on a per-case basis due to varied sampling performance by Rosetta. We set the dist_thresh to a lower value if decoy generation stage is able to provide high quality decoys (min_dist≤0.5Å). However, if Rosetta is unable to sample decoys closer to the native, we set the dist_thresh to higher values. Specifically, dist_thresh is set to 2Å for the easy cases (min_dist<1Å). For the medium cases (1Å≤min_dist<2Å), dist_thresh is either 2.5Å or 3Å. For the hard cases (3Å<min_dist), we increase the dist_thresh until one of the methods accumulates non-zero number of near-natives in the top selected basins. Moreover, if any test case belongs to a particular category based on the min_dist, but very few (for example, 10) near-natives are available in the decoy set according to that min_dist, we moved that test case to the next difficulty level. We calculated the knowledge-based features (potentials) used in the regression model for weighted decoy selection as follows. We calculated the *RW* potential in the form of *calRW* and *calRWplus* using publicly available executables [64]. The *dDFIRE* potential has been calculated using *dDFIRE* program [65]. A boosting-based ensemble learning approach, XGBoost [56], has been used to build the regression model. XGBoost is fast, scalable, and controls overfitting [66].

## 3. Results

While details behind the methodologies evaluated in this section can be found in Section 2, we summarize them here in the interest of clarity.

### 3.1. Evaluation Metrics

Methodological details aside, a method can be characterized as selecting (and offering for prediction) *a subset of decoys* or selecting *an individual decoy* from a given decoy dataset. In the former, such methods can be evaluated in terms of *purity*, a metric we originally introduced in [14]. In the latter, methods can be evaluated via *loss*, a classic machine learning (ML)metric that we adopt and propose here.

Let us suppose that methods of the first category, which include clustering-based methods, organize decoys in a given dataset into groups. These groups can be ranked/ordered based on characteristics that can be measured over a group. For instance, one such characteristic can be size. Ordering by largest to smallest can provide groups $G_{1},\ldots ,G_{n}$, with *n* being the total number of identified groups. Such a method that first organizes decoys into groups and then ranks them can be used for decoy selection as follows: Provided a user-specified parameter *l*, the groups $G_{1},\ldots ,G_{l}$ in the ranking $G_{1},\ldots ,G_{n}$ can be selected, and decoys in them offered as prediction of near-native structures. The selected set $G_{1},\ldots ,G_{l}$ can be evaluated in terms of its *purity*; that is, how many near-native decoys are actually contained in the selected set. On a test case, where the native structure is known, all given decoys (generated by a decoy generation algorithm) can be evaluated in terms of their dissimilarity from the native structure. We employ least root-mean-squared-deviation (RMSD), which averages the Euclidean distance among atoms in two given structures over the atoms after removing differences due to rigid translation and rotation in 3D [67]. Provided a distance threshold dist_thresh, all decoys below the threshold are labeled as near-native; the rest are labeled as non-native. The former are positives, and the latter are negatives. Thus, a selected set *S* of decoys (consisting of decoys in groups $G_{1},\ldots ,G_{x}$) can be evaluated in terms of its purity TP(S)/|S|, where TP(S) is the number of near-native decoys (true positives) in *S*. It is evident that purity is related to precision, a classic ML metric.

Methods in the second category select decoys directly. We note that one can easily put together a pipeline that follows up a method from the first category with a method from the second category. For instance, after selecting first a subset *S* of decoys from a given dataset, uniform random sampling can be employed to select any decoy from *S* and offer for prediction. We propose *loss* to evaluate how good a selected decoy is. The decoy that is closest to the native structure (in terms of RMSD) has a loss of zero. A perfect method would always find such a decoy. Let us refer to this decoy as *BestDecoy*. In the absence of such a method, any other selected decoy *SelectedDecoy* presents a loss measured as *RMSD (SelectedDecoy, NativeStructure)—RMSD (BestDecoy, NativeStructure)*.

It is worth noting that several metrics are available to assess the quality of a given decoy in comparison to a given native structure. In addition to the least RMSD (described above), template modeling (TM)-score and global distance test, total score (GDT-TS) are very popular. The score, GDT-TS, reports the average coverage of the target sequence of the substructures at 1, 2, 4, and 8 Å distance cutoffs [68]. The approaches used in GDT and another scoring function MaxSub [69] are extended by TM-score, and it eliminates the protein size-dependency in calculating the score for a random protein structure pairs [70]. Generally, a decoy with a TM-score 0.5 or higher is considered to be of very high quality (very similar to a given native structure to which it is compared via TM-score). It is reported in the range 0–1 in the results below, rather than using percentages. A decoy with a GDT-TS score of 0.4 or higher is considered to be of very good quality (generally indicating that a large fraction of amino acids are within some distance from a native structure under comparison).

### 3.2. Evaluation Setup

Let us summarize the methods evaluated below via purity or loss, depending on the category to which they belong. The baseline method groups decoys via the k-means clustering algorithm, as detailed in Section 2. While the clusters/groups this method identifies can be ranked/ordered via different characteristics, which we propose in recent work [14], for clarity of presentation, we focus only on size and evaluate, via purity, the largest cluster (with the largest number of decoys). The second method we evaluate here for the purpose of comparison also performs clustering but by leveraging network community detection methods. This method has been recently published [16].

These clustering methods, which we will refer to from now on as KMeans-Select and Community-Select, do not employ energies that are available for decoys generated by a decoy generation algorithm. To show the improved performance (in purity) when considering these energies in the context of the energy landscape, we evaluate here Basins-Select, a method recently published in [14] that organizes decoy-energy pairs in a graph over which it identifies basins and offers the largest basin (with the most decoys) for prediction.

All the above three methods implement unsupervised learning, as they organize decoys in clusters or basins without any prior knowledge. In contrast, the fourth method we present, ML-Select, leverages supervised learning to select basins of better quality. Effectively, the method trains itself over basins with associated purities on a subset of cases (different proteins) to learn to predict the purity of a basin on the rest of the cases. These predictions are employed to select basins of higher purity. All these methods are described in greater detail in Section 2.

As Section 2 relates, we propose Weighted-Decoy-Select, a novel method that is able to select individual decoys. Rather than relying on random sampling of decoys from a set, the method associates a weight with each decoy that reflects its quality and selects the decoy with the highest weight. Two variants of this method are implemented and evaluated based on two different definitions of decoy weight. As described above, this method (its two variants) is evaluated via loss.

### 3.3. Benchmark Datasets

We evaluated our method’s performance on two benchmark datasets. We first show results for 17 proteins of different lengths (number of amino acids) and folds, as shown in Table 1. For each of these protein targets, we use the target’s amino acid sequence and 3- and 9-residue fragments (generated by Robetta) as inputs to the Rosetta *template-free* decoy generation algorithm to generate between 50,000 and 68,000 decoys per target. The size of the decoy dataset |Ω| generated for each protein is listed in Column 6 in Table 1. The minimum RMSD (min_dist) between any decoy in the dataset of a target and the corresponding known native structure of the target (obtained from the PDB in the PDB entries shown in Column 1) are shown in Column 7. The categorization of the test cases into easy, medium, and hard has been determined based on min_dist, as related in Section 2.6.

Our second dataset consists of seven CASP targets selected from the free modeling category in CASP 12 and CASP 13 (see Table 2). We use the same procedure as described above to generate between 36,860 and 55,000 decoys per target. The size of the decoy dataset |Ω| generated for each CASP target is listed in Column 4 in Table 2. The minimum RMSD (min_dist) between any decoy in the dataset of a target and the corresponding known native structure of the target, see Column 5.

### 3.4. Learning over Landscapes Yields Purer Decoy Subsets

**Figure 2:**
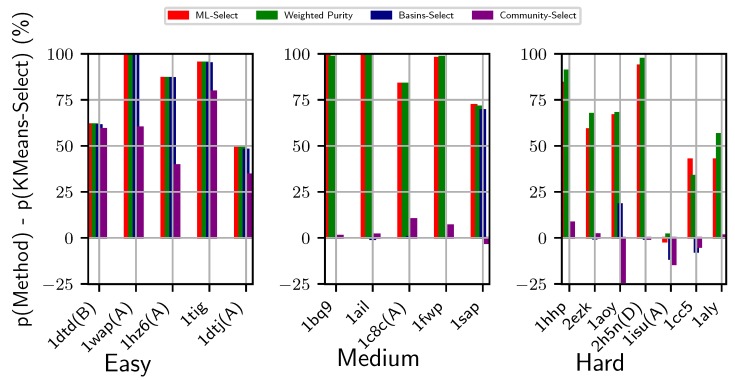
Visualization of two supervised and two unsupervised decoy selection strategies. The *y*-axis tracks the difference in purity of the top basin predicted by each method with the purity of the top (largest) cluster obtained by KMeans-Select. The *x*-axis tracks the PDB entry id of each target protein. Color-coding is used to distinguish the different methods under comparison. The left, middle, and right panels show results for the easy, medium, and hard datasets, respectively.

Figure 2 provides a visual comparison of energy-based and energy-less, unsupervised and supervised decoy selection methods, namely Basins-Select, ML-Select, and Community-Select, against the baseline method KMeans-Select on non-CASP dataset shown in Table 1. Specifically, we show how each method fares against KMeans-Select by subtracting the purity achieved by KMeans-Select from each method’s purity. Results are presented for each category (easy, medium, and hard), separately, in the left, middle, and right panels, respectively, in Figure 2. Weighted Purity, described in Section 2, is also shown.

Figure 2 shows that, while the basin-based selection methods (Basins-Select, ML-Select, and Weighted Purity) perform comparably on the easy datasets, Community-Select underperforms in 3/5 of these datasets. For instance, for the protein under PDB entry 1hz6(A), Community-Select achieves only 39.5% purity, whereas the basin-based methods achieves more than 85% purity (after deducting the baseline method KMeans-Select’s top cluster purity). The supervised methods (ML-Select and its Weighted Purity) outperform others on the medium-difficulty and hard datasets. In particular, the benefit of considering energy in the context of the energy landscape for grouping decoys into basins for decoy selection becomes prominent on the medium-difficulty datasets. Furthermore, ML-Select (and its Weighted Purity) outperforms the unsupervised energy-based selection method (Basins-Select) and energy-less clustering-based method (Community-Select) on all the medium-difficulty datasets. While Basins-Select performs well on 1/5 of the datasets (with corresponding PDB entry 1sap), Community-Select performs worse than the baseline (KMeans-Select) on this dataset.

The merit of energy landscape-based supervised learning becomes more prominent on the hard datasets. Specifically, ML-Select significantly outperforms the remaining selection methods and is supported by Weighted Purity on 6/7 of the hard datasets. More importantly, the supervised learning model perform consistently well for all the test cases regardless of their difficulty levels. The inadequacy of energy-less clustering method Community-Select is more visible on the hard datasets. For instance, Community-Select performs significantly worse than the baseline method KMeans-Select for the protein under PDB entry id 1aoy (25.7% purity of top cluster obtained by KMeans-Select against 0.58% purity of top cluster/community obtained by Community-Select).

**Figure 3:**
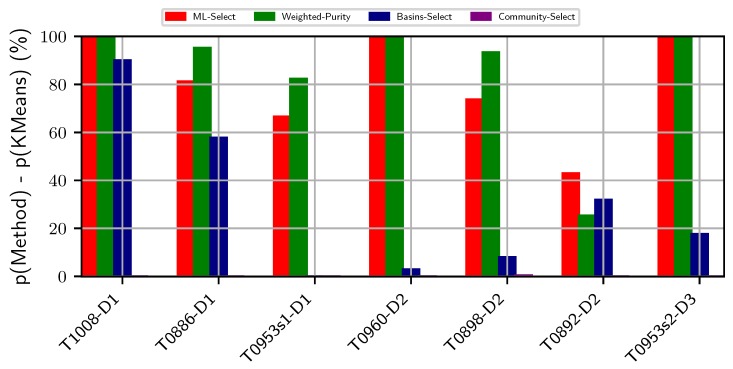
Visualization of two supervised and two unsupervised decoy selection strategies on the critical assessment of structure prediction targets. The *y*-axis tracks the difference in purity of the top basin predicted by each method with the purity of the top (largest) cluster obtained by KMeans-Select. The *x*-axis tracks the protein data bank entry id of each target protein. Color-coding is used to distinguish the different methods under comparison.

Figure 3 compares purity obtained by the two supervised and two unsupervised methods on the CASP targets. Community-Select performs poorly on all seven targets. Specifically, this method fails to provide more than 0% percent purity on five out of seven targets and obtains less than 1% purity on the remaining two targets. Basins-Select performs reasonably well on two targets (T1008-D1 and T0886-D1). However, it performs similarly to Community-Select on 4/7 targets. In contrast, ML-Select obtains high purity (even reaching 100%) on 6/7 of the CASP targets. ML-Select’s consistent success in obtaining good purity results on both non-CASP and CASP targets demonstrates its utility in selecting decoy subsets of good quality.

It is worth noting that the utility of Weighted Purity lies in its ability to inform about the overall quality distribution of decoys in a selected basin. As related in Section 2.5, the purity metric assumes a uniform distribution and treats each decoy in the selected basin as of similar quality, which does not always conform with the real-life scenario. Practically, a basin or a cluster comprise decoys of varying qualities. Selecting a decoy blindly at random from the top cluster or basin does not guarantee the best decoy available in the basin. As we set a threshold to determine the near-natives, two near-native decoys might be in varying distances from the native structure albeit below the pre-defined threshold. The weights assigned to each decoy in the selected top basins expose this hidden information of varying decoy qualities regarding how far away they are from the native structure. By taking into account the weight (or confidence level) of each decoy, the expected purity metric offers a holistic quality measure of a selected basin. Two basins with the same purity values may score two different expected purity values, which would inform about the quality of the decoys populating the two basins at hand. For instance, as shown in Figure 2, the expected purity (indicated by Weighted Purity) of protein with PDB entry 2h5n(D) is higher than its purity measure, whereas the expected purity of protein with PDB entry 1cc5 is lower than its purity measure. One can interpret this result as the top basin for protein with PDB entry 2h5n(D) consisting of much higher quality decoys than does the top basin of protein with PDB entry 1cc5. As shown in Figure 3, similar performance is observed over the CASP targets. Specifically, the expected purity of the CASP target T0898-D2 is higher than its purity measure. In contrast, the purity result of T0892D2 is higher than its expected purity. As such, expected purity would prove useful in deciding on which basin to prefer for selecting a decoy for further analysis.

### 3.5. Learning-Based Selection of Decoys Yields Lower Loss

We now relate the performance of Weighted-Decoy-Select, which selects one individual decoy from the top basin obtained by ML-Select. As detailed in Section 3, two variants of this method are considered, based on two options in associating weights with decoys in a basin. The first variant, which we refer to as Weighted-Decoy-Select${}_{\mathrm{predicted}-\mathrm{rmsd}}$, associates weights that are the inverse of RMSDs predicted via regression, as detailed in Section 2.5. The second variant, which we refer to as Weighted-Decoy-Select${}_{\mathrm{density}-\mathrm{based}}$, associates weights based on a density score, also detailed in Section 2.5.

Table 3 relates the RMSD loss described above in Section 3.1 for each variant in Columns 2 and 3, respectively. In comparison, the loss obtained by a method that selects a decoy uniformly at random (which we refer to as Random-Decoy-Select in Table 3) is shown in Column 4. We note that Random-Decoy-Select is repeated 10 times, and Column 4 relates the average RMSD loss. To place these results in context, the median RMSD (over all decoys in a dataset) from the known native structure is shown in Column 5, the percentage of decoys with RMSDs less than 3Å from the known native structure is shown in Column 6, and the percentage of decoys with RMSDs less than 1.5 Å from the best decoy (closest to the known native structure) is shown in Column 7.

As shown in Table 3, on 7/24 of the datasets (17 non-CASP targets and seven recent CASP targets), the RMSD loss is <0.5 Å; on 11/24 of the datasets, the loss is subangstrom. The loss from Random-Decoy-Select is the same or greater than the loss from the weighted variants, albeit with small differences. This is due to the fact that the basins obtained by ML-Select are of very high quality to begin with, and even a uniform random sampling of decoys over the top basin provides good decoys. It is worth noting that the scarcity of good decoys, as evident in Column 6 and Column 7, reveals the main challenge behind decoy selection for template-free protein structure prediction: 11/24 of the decoy datasets contain no decoys closer than 3Å to the native structure; 17/24 of the datasets contain less than 1% of decoys below 1.5Å away from the best decoy in the dataset. These statistics clearly relate that decoy selection is a needle(s)-in-the-haystack problem. In light of these statistics, the results obtained by our decoy selection pipeline ML-Select→Weighted-Decoy-Select are highly encouraging.

### 3.6. Comparison with State-of-the-art Decoy Selection Methods on Critical Assessment of Structure Prediction Targets

Table 4 compares TM-score loss and GDT-TS loss due to ML-Select with that of three state-of-the-art model quality estimation methods, MUFOLD-CL, Qprob, and SBROD. MUFOLD-CL is a multi-model (clustering-based) method that clusters the decoys and then selects cluster representatives [71]. Qprob [72] and SBROD [73] are single model methods. As shown in Table 4, ML-Select obtains lower losses for most of the CASP targets.

Execution time varies per method. Qprob takes the longest to finish. On a dataset of 55,000 decoys (T0960-D2), Qprob takes 12 h, 41 min, and 55 s to finish execution. On the same dataset, SBROD takes 8 h, 39 min, and 25 s. MUFOLD-CL takes 26 min and 18 s. ML-Select’s execution time varies depending on whether it is running on a dataset for the first time. The total execution time including the identification of basins on a dataset of 55,000 decoys (T0960-D2) is 1 h, 1 min and 25 s. Subsequent executions take only 9.3 s. Similarly, Basins-Select takes 1 h, 1 min, and 16 s to finish execution for the first time on the same dataset. Subsequent executions require 16 s. Community-Select takes a substantial amount of time to finish on the same dataset, requiring 6 h, 42 min, and 12 s.

### 3.7. Detailed Analysis: Distribution of Decoys Affects Method Performance

**Figure 4:**
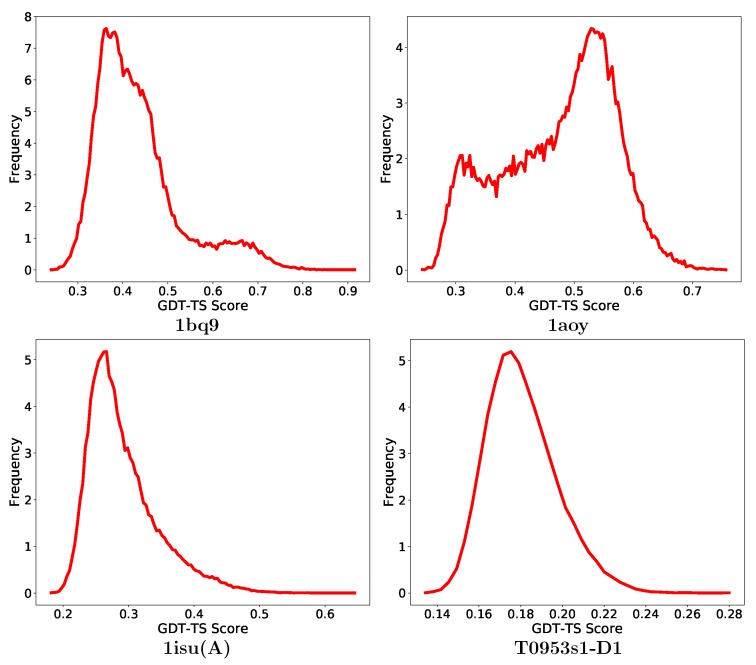
Distribution of global distance test-total score scores for one medium difficulty protein under PDB entry 1bq9, two hard proteins under PDB entries 1aoy and 1isu(A) from the non-CASP targets and T0953s1D1 from the CASP targets. The *x*-axis tracks GDT-TS scores. The *y*-axis tracks frequency of decoys with corresponding GDT-TS scores. The frequency has been scaled to a range [1–10] by dividing all frequencies by the minimum frequency.

Here, we look deeper for the impact of the quality of the decoys on the performance of decoy selection. Figure 4 shows the distribution of GDT-TS scores of decoys in one CASP target, and one medium, and two hard targets selected from the non-CASP list of proteins. As presented in Figure 4, the GDT-TS distribution for protein with PDB entry 1bq9 shows a congregation of mostly low-quality decoys. A grouping-based method that only emphasizes on the size of a group when selecting a decoy subset may select this low-quality decoy group and so report low purity. Similarly, the GDT-TS distribution of the protein under PDB entry 1aoy shows no clear grouping of good-quality decoys, which makes it hard to identify a good-quality group. In both cases, ML-Select is able to overcome these challenges and obtain high-purity results. The GDT-TS distribution of protein with PDB entry 1isu(A) and the CASP target T09523s1D1 represent difficult decoy sets. As shown in Figure 4, their GDT-TS distributions show one large group of low-quality decoys which may mislead grouping-based methods to select this group or a part of it for prediction. Although ML-Select performs fairly well on the decoy set of T0953s1D1 (more than 66% purity), all methods, including ML-Select, struggle on the decoy set of 1isu(A) and fail to provide a decoy subset with satisfactory purity.

## 4. Discussion and Conclusions

The results presented here suggest that leveraging the energy landscape probed by a template-free PSP method for decoy selection is promising and warrants further investigation. While energy has long been justifiably ignored in favor of structural similarity for identifying near-native decoys, the work presented here shows that energy can be reliably employed to find near-native decoys.

Observations on methods based on clustering reveal that these methods fail to provide a reasonably good performance, which is attributed to the decoy dataset not being tightly bound. It is not uncommon in such cases that the near-native decoys are few and far away from the rest of the decoys. As a result, clustering methods, seeking consensus, are at a disadvantage. As demonstrated here, the energy landscape treatment of decoy selection is promising in such cases. Specifically, basins in the energy landscape can prove instrumental in obtaining a subset of good-quality decoys, as well as individual decoys of good quality.

Specifically, this paper presents a pipeline that integrates unsupervised and supervised learning. Energy basins extracted from the landscape serve as the building block of the pipeline. The supervised learning component of the pipeline, ML-Select, utilizes energy- and graph-based characteristics of basins and successfully identifies good-quality basins even for exceptionally challenging decoy datasets. The evaluation of the quality of predicted basins considers the number of false positives and heavily penalizes a basin that contains more false positives (non-native decoys) than true positives (near-native decoys). Hence, when narrowing the focus to high-quality basins, even a decoy selected uniformly at random over the decoys in a basin is more likely than not a near-native, which is reflected in the related results. While for each dataset the decoy selected by the weighted selection method is closer to the native structure than a randomly-selected decoy, the performance difference is small in most cases. This phenomenon indicates that ML-Select selects basins of high quality.

The presented pipeline also offers a probabilistic estimate of the precision of quality of the selected basins, while indirectly informing about the quality of decoys populating those basins. Such an estimate offers a distribution of decoy quality in a basin which may prove helpful in choosing a basin when offered multiple basins as prediction. A well-known limitation of consensus-based method is that they are not able to provide an absolute global quality of single decoys in a cluster/group. The Weighted-Decoy-Select method and the resulting probabilistic estimate of precision of the selected basins offer a solution to this limitation.

Although the ML-based pipeline presented in this paper offers a promising solution to decoy selection in template-free PSP, further investigation is warranted, particularly, on weighting schemes that can better distinguish between good and better decoys. It is still an unresolved question as to what characteristics define the best decoy in a good basin and distinguish it from, say, the focal minimum. Future work will consider the incorporation of more features, not necessarily energy-based, to possibly improve the quality of predictions. The line of inquiry pursued in this paper presents a promising direction for advancing decoy selection. We further explore other methods ([74,75]) to improve the decoy selection.

## Figures and Tables

**Table 1:** Testing dataset (* denotes proteins with a predominant β fold and a short helix). Protein Data Bank entries of corresponding known native structures are shown in Column 3. Folds of native structures are shown in Column 4. The length of each sequence (number of amino acids) is shown in Column 5. The size of the Rosetta-generated decoy dataset (number of decoys) is shown in Column 6. The lowest root-mean-squared-deviation to the known native structure in each dataset is shown in Column 7. As described above, based on this proximity, the datasets are categorized into easy, medium-difficulty, and hard.

Difficulty	#	PDB Entry	Fold	Length	|Ω|	min_dist
						(Å)
Easy	1	1dtd(B)	α+β	61	58,745	0.51
	2	1wap(A)	β	68	68,000	0.68
	3	1hz6(A)	α+β	64	60,000	0.69
	4	1tig	α+β	88	60,000	0.70
	5	1dtj(A)	α+β	74	60,500	0.74
Medium	6	1bq9	β	53	61,000	0.98
	7	1ail	α	70	58,491	1.01
	8	1c8c(A)	$\beta^{*}$	64	65,000	1.04
	9	1fwp	α+β	69	51,724	1.63
	10	1sap	β	66	66,000	1.93
Hard	11	1hhp	$\beta^{*}$	99	60,000	1.85
	12	2ezk	α	93	54,626	2.89
	13	1aoy	α	78	57,000	3.03
	14	2h5n(D)	α	123	54,795	3.46
	15	1isu(A)	coil	62	60,000	3.67
	16	1cc5	α	83	55,000	4.31
	17	1aly	β	146	53,000	9.38

**Table 2:** Dataset collected from critical assessment of protein structure prediction data archive. The target IDs are shown in Column 2. The length of each sequence (number of amino acids) is shown in Column 3. The size of the Rosetta-generated decoy dataset (number of decoys) is shown in Column 4. The lowest RMSD to the known native structure in each dataset is shown in Column 5.

#	Target ID	Length	|Ω|	min_dist
				(Å)
1	T1008-D1	77	55,000	1.54
2	T0886-D1	69	55,000	4.92
3	T0953s1D1	67	55,000	5.81
4	T0960-D2	84	55,000	5.98
5	T0898-D2	55	43,435	6.0
6	T0892D2	110	36,860	6.62
7	T0953s2D3	77	55,000	7.52

**Table 3:** Columns 2–4 relate the loss obtained from Weighted-Decoy-Select${}_{\mathrm{predicted}-\mathrm{rmsd}}$, Weighted- Decoy-Select${}_{\mathrm{density}-\mathrm{based}}$, and Random-Decoy-Select, respectively. Columns 5–7 show the median RMSD (over all decoys in a dataset) from the known native structure, the percentage of decoys in each dataset with RMSDs less than 3Å from the known native structure, and the percentage of decoys with RMSDs less than 1.5Å from the best decoy (closest to the known native structure). The last seven rows show results for the CASP targets. Lowest loss per target is highlighted in bold.

	Loss (Å)					
Targets	Weighted-Decoy-Select${}_{\mathrm{predicted}-\mathrm{rmsd}}$	Weighted-Decoy-Select${}_{\mathrm{density}-\mathrm{based}}$	Random-Decoy -Select	Median RMSD (Å)	< 3Å (%)	< min_dist + 1.5Å (%)
1hz6(A)	0.7	0.47	0.5	3.6	24.8%	11.1%
1dtj(A)	0.26	0.36	0.51	7.94	4%	3.4%
1tig	0.46	0.13	0.4	8.24	4.2%	2.4%
1dtd(B)	0.36	0.66	0.51	10.36	3.1%	2.34%
1wap(A)	0.46	0.46	0.46	11.58	0.54%	0.51%
1ail	0.5	0.56	0.8	8.32	1.5%	0.57%
1bq9	1.9	1.9	1.9	9.1	0.29%	0.06%
1sap	0.98	0.7	0.7	9.57	7.02%	11.57%
1fwp	0.27	0.27	0.27	9.92	0.38%	0.3%
1c8c(A)	0.54	0.64	0.7	10.0	6.8%	3.03%
2ezk	2.3	2.3	2.5	8.71	0.2%	0.004%
1aoy	4.8	4.4	5.0	9.16	0%	0.15%
1cc5	4.1	3.7	4.2	10.75	0%	0.4%
1isu(A)	5.3	6.6	6.3	10.77	0%	0.02%
2h5n(D)	0.43	1.0	1.5	13.61	0%	0.04%
1hhp	12.4	12.4	12.4	14.74	0.03%	0.03%
1aly	3.1	3.8	4.7	17.57	0%	0.02%
T1008-D1	0.56	0.56	1.2	9.5	0.87	0.95%
T0886-D1	4.5	4.6	6.6	12.12	0%	0.04%
T0953s1D1	4.9	4.9	6.0	12.6	0%	0.05%
T0960-D2	2.8	2.8	2.8	11.2	0%	0.28%
T0898-D2	3.6	2.9	4.1	10.33	0%	1.6%
T0892D2	7.1	3.7	7.6	13.4	0%	0.15%
T0953s2D3	2.8	2.8	2.8	12.21	0%	0.23%

**Table 4:** Columns 2–6 relate template modeling-score and global distance test-total score loss obtained from Weighted-Decoy-Select${}_{\mathrm{predicted}-\mathrm{rmsd}}$, Weighted-Decoy-Select${}_{\mathrm{density}-\mathrm{based}}$, MUFOLD-CL, Qprob, and SBROD, respectively. Lowest loss per CASP target is highlighted in bold.

	TM-Score Loss, GDT-TS Loss				
Target ID	Weighted-Decoy-Select${}_{\mathrm{predicted}-\mathrm{rmsd}}$	Weighted-Decoy-Select${}_{\mathrm{density}-\mathrm{based}}$	MUFOLD-CL	Qprob	SBROD
T1008-D1	0.009, 0.019	0.010, 0.016	0.009, 0.019	0.013, 0.016	0.007, 0.016
T0886-D1	0.011, 0.014	0.011, 0.014	0.019, 0.026	0.016, 0.033	0.014, 0.025
T0953s1D1	0.018, 0.029	0.011, 0.026	0.017, 0.019	0.022, 0.037	0.019, 0.019
T0960-D2	0.010, 0.018	0.010, 0.018	0.020, 0.012	0.016, 0.021	0.018, 0.015
T0898-D2	0.010, 0.018	0.010, 0.027	0.007, 0.027	0.013, 0.023	0.020, 0.032
T0892D2	0.018, 0.016	0.016, 0.018	0.032, 0.025	0.017, 0.018	0.013, 0.016
T0953s2D3	0.014, 0.023	0.014, 0.023	0.024, 0.032	0.025, 0.032	0.028, 0.032

## Abbreviations

The following abbreviations are used in this manuscript:

PDB	Protein Data Bank
CASP	Critical Assessment of protein Structure Prediction
lRMSD	Least Root-Mean-Squared-Deviation
ML	Machine Learning
NN	Neural Network
RF	Random Forest
PC	Pareto Count
PR	Pareto Rank
SVM	Support Vector Machines
NSF	National Science Foundation
LDRD	Laboratory Directed Research and Development
